# Serum Hepcidin Level as a Marker of Iron Status in Children with Cystic Fibrosis

**DOI:** 10.1155/2018/3040346

**Published:** 2018-07-02

**Authors:** Monika Kałużna-Czyż, Urszula Grzybowska-Chlebowczyk, Halina Woś, Sabina Więcek

**Affiliations:** ^1^Department of Paediatrics, School of Medicine in Katowice, Medical University of Silesia, Katowice, Poland; ^2^Department of Nursing and Emergency Medicine, Faculty of Health Sciences, University of Bielsko-Biała, Bielsko-Biała, Poland

## Abstract

**Introduction:**

Iron deficiency is common in patients with cystic fibrosis. Conventional iron status markers are often abnormal in patients with CF, reflecting inflammation and/or infection, rather than actual iron stores. The aim was to evaluate serum hepcidin levels against selected iron status markers, assuming that hepcidin may be a more sensitive indicator of iron management in patients with active inflammation, such as those with CF.

**Material and Methods:**

46 children with cystic fibrosis and 31 healthy controls were enrolled. Hepcidin concentration was evaluated, along with the following other blood assays: full blood count, Fe, ferritin, transferrin, TIBC, liver markers, and CRP.

**Results:**

Higher ferritin and CRP levels as well as lower TIBC levels significantly predicted hepcidin levels in the study group, control group, and the entire sample. There was no significant difference in hepcidin levels between the patients and controls. Children with exacerbations had significantly higher hepcidin levels than those with stable disease. These findings support the serum hepcidin level as useful in assessing iron status in children with cystic fibrosis. It may also be useful in early detection and monitoring of treatment of exacerbations.

## 1. Introduction

Children with cystic fibrosis (CF) are at high risk of iron deficiency (ID). Chronic inflammation, concomitant gastrointestinal conditions, and malnutrition often seen in CF make these patients particularly susceptible to ID. The WHO defines ID as a ferritin concentration below 12 mg/L in children below 5 years of age and below 15 mg/L in children above 5 years of age [1]. The incidence of iron deficiency in patients with CF is estimated from 33% in the paediatric population to over 60% in adult patients [2–7]. More specifically, the reports from the 1980s estimate the prevalence of iron deficiency in children and young people with CF aged 9 weeks to 25 years at 32% and those aged 1.5 month to 22 years at 33% [7, 8]. A more recent study in the paediatric CF population estimates the prevalence of iron deficiency at 17% and iron deficiency anaemia to 11.3% [2]. In this study with a long-term follow-up of 14 years, iron deficiency was diagnosed in 60.4% of enrolled children in at least one year [2]. Although ID in patients with CF has been relatively widely quantified, the clinical consequences of ID in children with cystic fibrosis are not fully known. Therefore, more research data is needed to expand our knowledge of clinical consequences of abnormal iron status in CF.

Conventional markers of iron status are often abnormal in patients with cystic fibrosis, reflecting inflammation and/or infection rather than the actual iron deficiency. Ferritin, for instance, is an acute phase protein, which is elevated during the inflammation. As a result, using these markers is associated with a high risk of false positive results (75%) but also of false negative results [9]. Difficulties in diagnosis and differentiation of iron deficiency in these patients support the need to seek new, more sensitive, and more specific markers of iron status [9].

Until recently, the soluble transferrin receptor (sTfR) was the best parameter in assessing iron deficiency in cystic fibrosis, based on which the sTfR (*μ*g/mL) to the logarithm of serum ferritin (mg/L) (TfR/logFer) ratio is calculated, as these values are not inflammation-dependent [10]. However, Uijterschout et al. demonstrated that sTfR is not useful in diagnosing iron deficiency in relatively healthy children with cystic fibrosis, since it is only elevated in response to a sudden decrease in body iron stores, whereas such rapid changes to the iron status do not occur in children with cystic fibrosis. A significantly higher sTfR/logFer ratio seen in children with iron deficiency is probably associated with lower ferritin levels [11].

The first report on the use of hepcidin was published in 2000 [12]. Hepcidin is a hormone which plays the major role in maintaining iron homeostasis in human body [13, 14]. Synthesized in hepatocytes by interleukin-6 (IL-6) and lipopolysaccharides (LPS), it constitutes a part of the body defence system during an infection, ensuring that iron stores are kept out of reach of the microbes. Hepcidin levels are elevated in response to an infectious agent, which reduces intestinal iron absorption and iron recovery by macrophages; acting on erythrocytes and macrophages, hepcidin inhibits iron recycling from reticuloendothelial cells [15, 16]. As an acute phase protein, hepcidin, along with ferritin, is an inflammatory marker, but its level decreases much faster during the healing phase [17]. There is indirect evidence supporting the presence of this nonspecific defence mechanism, which affects iron metabolism, also in children with cystic fibrosis.

In the medical literature, there is insufficient data on hepcidin in patients with cystic fibrosis, as well as on possible diagnostic and therapeutic implications of such research. Understanding the role and effect of hepcidin on iron homeostasis in this population might contribute to the development of new diagnostic and therapeutic options in future. So far, a few reports have been published, which evaluate hepcidin levels mainly in adults with cystic fibrosis [18, 19]. It was only in 2014 that Uijterschout et al. published their study on the value of hepcidin in the assessment of iron status in children with cystic fibrosis [11]. They concluded that hepcidin could be used as an early indicator of deficient iron stores in children with CF. Indeed, inflammation and infection associated with elevated CRP levels lead to an increased hepcidin synthesis in hepatocytes [20]. Therefore, hepcidin may theoretically be a more sensitive indicator of the iron status in patients with active inflammation, including children with cystic fibrosis. The literature additionally emphasizes that the relationship between hepcidin and CRP is stronger in paediatric population than in adults [20]. Hence, a stronger impact of inflammation/infection on hepcidin levels can be expected in children than in adults.

The aim of this study was to evaluate the serum hepcidin levels against the selected parameters of iron status such as ferritin, transferrin, serum iron levels, and TIBC (*total iron-binding capacity* which is more often used clinically than serum transferrin assay) in children with cystic fibrosis and healthy controls. It was hypothesized that hepcidin may be a more sensitive iron status marker than conventionally assessed indicators in patients with active inflammation, such as those with CF.

## 2. Material and Methods

The study was conducted in the Department of Paediatrics, Medical University of Silesia and the Cystic Fibrosis Outpatient Clinic of the Upper Silesian Child Health Centre in Katowice. The study protocol was approved by the Bioethics Committee by the Medical University of Silesia in Katowice (KNW/0022/KB1/138/11). A total of 77 patients—44 boys (57%) and 33 girls (43%), at the age of 6 months to 18 years (mean age of 6.85 years) were enrolled.

The study group consisted of 46 patients with cystic fibrosis diagnosed based on history, clinical symptoms, and elevated sweat chloride levels. The molecular study of CFTR gene additionally confirmed the diagnosis in this group. The following exclusion criteria were applied to the study group: any comorbidities which may affect haematopoiesis and/or iron status (e.g., cancer, juvenile rheumatoid arthritis, endocrine disorders, and hemochromatosis). Patients receiving iron supplements were also excluded. The study subjects were later subdivided by their exacerbation status into the acute and stable subgroups. The acute subgroup consisted of patients with the exacerbation of cystic fibrosis on enrolment, whereas the stable subgroup consisted of patients in the stabilization phase on enrolment. The exacerbation criteria included elevated inflammatory markers (CRP > 10 mg/L) and/or worsening of bronchopulmonary disease, which required antibiotic treatment within one month prior to enrolment [11]. The control group consisted of 31 healthy children. Children with a history of infection within 4 weeks prior to enrolment, children born prematurely, and children receiving iron supplements within 6 weeks prior to enrolment were excluded. All participants and their parents/legal guardians were informed of the purpose and scope of the research, and informed consent was obtained in writing from parents/legal guardians as well as young people over 16 years of age.

The baseline assessment in both groups included the following: medical history, physical examination, nutritional status (BMI *z*-score), full blood count, pancreatic markers, liver markers, as well as serum hepcidin, iron, ferritin, transferrin, TIBC, and TFS *(transferrin saturation)* levels. Additionally, in patients with cystic fibrosis, clinical manifestation and its current severity were assessed using spirometry (FEV1 in particular), radiograms, *Pseudomonas aeruginosa* colonisation status, and the presence of comorbidities (diabetes, liver, and biliary diseases). Spirometry was performed in 14 (30%) patients; its use was limited by patient age (above 6 years of age) as well as their ability and willingness to cooperate with the researcher during the assessment. Lung X-rays were obtained for 28 (61%) children.

Serum hepcidin levels were determined using DRG® Hepcidin 25 bioactive ELISA assay (DRG Instruments GmbH, Marburg, Germany). Its hepcidin detectability range is 0.35 ng/mL to 80 ng/mL. A fasting venous blood sample of less than 0.5 cm^3^ was collected from each study participant. Nonhaemolysed serum was prepared directly after blood collection, frozen, and stored at −20°C until analysed. Subsequently, serum samples were thawed at room temperature.

Iron deficiency (ID) was diagnosed based on serum ferritin levels decreased below 12 mg/L in children under 5 years of age and below 15 mg/L in children over 5 years of age, according to the criteria of the World Health Organization (WHO) [1]. Anaemia was defined as the serum haemoglobin (Hb) level of 2 or more standard deviations (SDs) below the mean level expected for age and sex [21]. Iron deficiency anaemia (IDA) was defined as anaemia associated with ID.

The data was processed using the Statistica bundle (StatSoft, Poland). The between-group differences were determined using the *t*-test for normally distributed variables or the Mann–Whitney *U* test and Kruskal-Wallis test for the variables, where the normality of distribution assumption was violated. The association between the variables was determined using the Spearman correlation coefficient. The frequency of nonparametric variables was calculated and compared depending on the size of subgroups and expected values using the chi-square test (for values above 5) or Fisher's exact test (for values below 5). Sensitivity, specificity, and cutoff values of hepcidin assay in CF population were determined using ROC analysis. The *p* value of <0.05 was considered statistically significant for all comparisons.

## 3. Results

The aim of this study was to evaluate the serum hepcidin levels against the selected parameters of iron status (ferritin, transferrin, and serum iron levels) in children with cystic fibrosis and healthy controls. A correlational study was designed with additional between-group comparisons between patients and controls, as well as subgroups within the study group, determined based on *P. aeruginosa* colonisation status and exacerbation versus stable disease.

The serum iron level below the lower reference threshold was demonstrated in 18 (39%) patients from the study group and 4 (13%) controls. Iron deficiency anaemia was found in 2 (4%) patients from the study group (boys aged 10 months and 17 years). There was a significant difference in the mean serum iron level between the study group and the control group (58.4 ± 28.2 *μ*g/dL versus 94.5 ± 38 *μ*g/dL, *p* < 0.05).

Low ferritin concentration (mean of 6.7 *μ*g/mL) in keeping with iron deficiency was observed in 2 (4%) patients (boys aged 9 and 21 months). It was lower than the mean ferritin concentration in the remaining patients in the study group (17.59 *μ*g/mL). There was no significant difference in the mean serum ferritin level between the study group and the control group (54.5 ± 57.8 mg/dL versus 80.2 ± 45.5 mg/dL, *p* < 0.05). Similarly, there was no significant difference in the mean serum hepcidin level between the study group and the control group Ranges, means, and standard deviation (SD) values for serum hepcidin levels in study group patients and controls are shown in Table 1.

**Figure 1:**
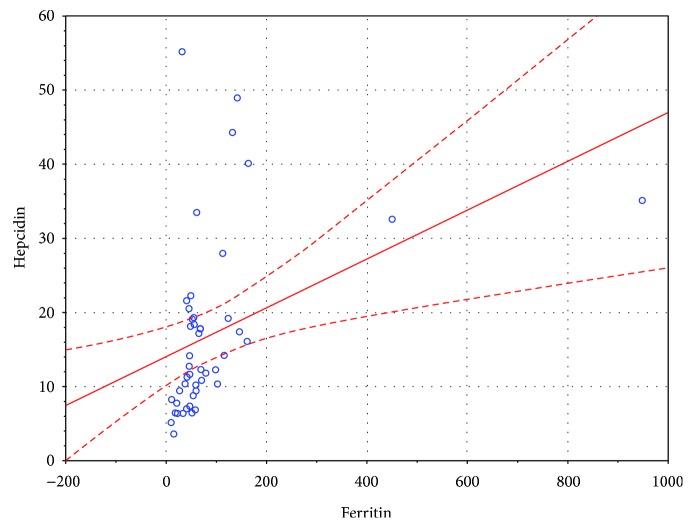
Correlation between serum hepcidin and ferritin levels in the study group.

**Figure 2:**
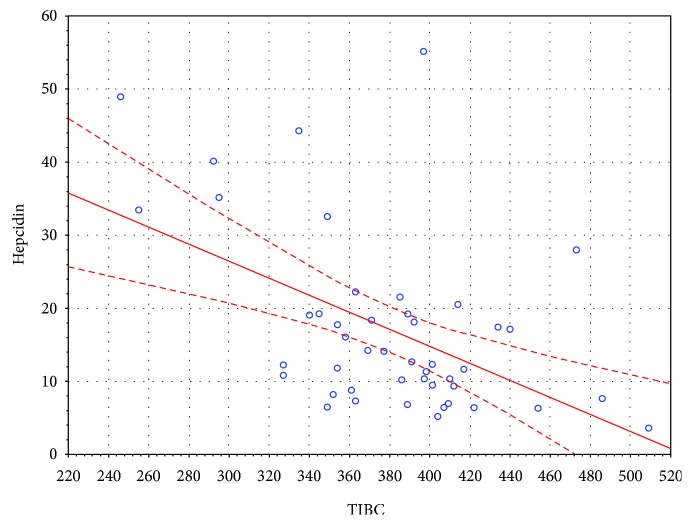
Correlation between serum hepcidin and TIBC levels in the study group.

**Figure 3:**
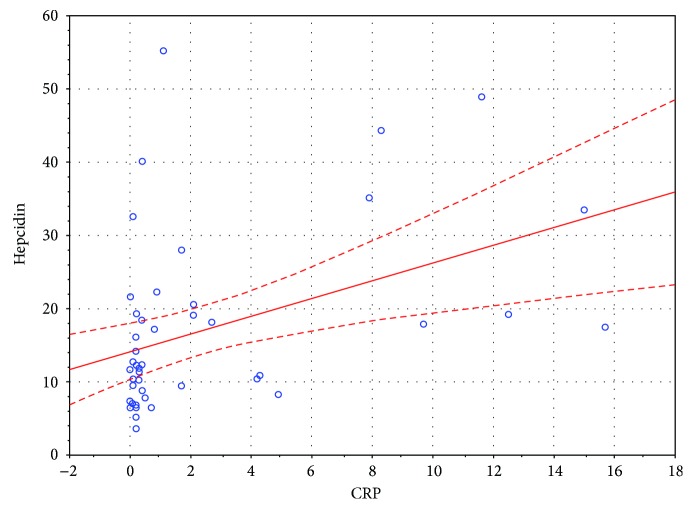
Correlation between serum hepcidin and CRP levels in the study group.

There was a significant positive correlation between serum hepcidin and ferritin levels in the entire sample, as well as in the study and control groups separately, *r*(75) = 0.55, *p* < 0.05; *r*(44) = 0.58, *p* < 0.05; and *r*(29) = 0.56, *p* < 0.05, respectively. There was a significant negative correlation between serum hepcidin and TIBC levels in the entire sample, as well as in the study and control groups separately, *r*(75) = −0.34 *p* < 0.05; *r*(44) = −0.46, *p* < 0.05; and *r*(29) = −0.38, *p* < 0.05, respectively. Furthermore, there was a significant positive correlation between serum hepcidin and CRP levels in the entire sample, as well as in the study and control groups separately, *r*(75) = 0.42, *p* < 0.05; *r*(44) = 0.46, *p* < 0.05; and *r*(29) = 0.36, *p* < 0.05, respectively. The nature of these associations in the study group was shown in Figures 1–3, respectively. There was no association between serum hepcidin levels and age, gender, weight, BMI, genotype, RBC-related full blood count parameters (RBC, Ht, Hb, MCV, MCH, MCHC, and RDW), or functional parameters indicative of liver (albumin), pancreas (WCS), and lung (FEV1) function.

**Figure 4:**
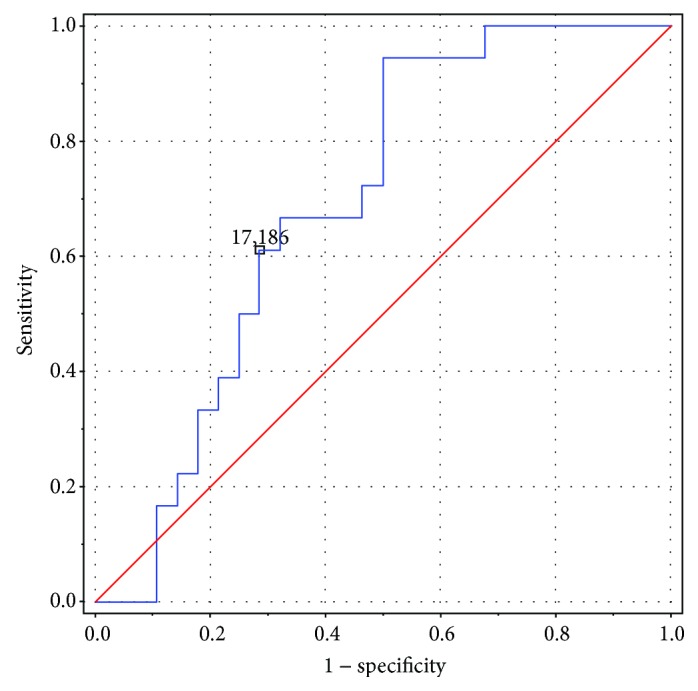
ROC curve.

Within the study group, comparisons were made between children with exacerbations and children with stable disease. There was no difference in serum iron, ferritin, transferrin, and TIBC levels, and full blood count or biochemical parameters between the two subgroups. However, children with exacerbations had significantly higher serum hepcidin levels than children with stable disease (19.22 ± 9.35 versus 15.7 ± 13.51, *p* < 0.05). The hepcidin cutoff value for the risk of exacerbations was determined using ROC curves. ROC metrics for serum hepcidin levels in the study group are shown in Table 2. The ROC curve for serum hepcidin levels in the study group is shown in Figure 4.

The study group subdivision based on the *P. aeruginosa* colonisation status showed no significant differences in any of the studied markers between the two subgroups.

## 4. Discussion

The study analysed the concentration of hepcidin in the blood serum of children with cystic fibrosis and healthy volunteers with particular emphasis on its role in the iron management. Higher ferritin and CRP levels as well as lower TIBC levels significantly predicted hepcidin levels in the study group, control group, and the entire sample. The correlations are not specific to cystic fibrosis, so they are potentially not caused by or linked to inflammation as they were also confirmed in healthy individuals [22]. The association between ferritin and hepcidin is consistent with the fact that the major action of hepcidin involves reducing iron absorption, whereas the increasing body iron stores will be reflected in an elevated ferritin level, as reported by Uijterschout et al. [11, 20]. The negative correlation between TIBC and hepcidin is in keeping with current understanding of the role of hepcidin as iron stores regulator and corresponds to observed increase of TIBC levels in iron deficiency.

The hypothesis of hepcidin level elevation in children with cystic fibrosis was only partly confirmed. An increase in hepcidin synthesis in response to the inflammation (exacerbation) was indeed shown, but there were no significant differences in hepcidin levels between study patients and healthy controls. However, within the study group, there was a significant difference in hepcidin levels between a subgroup of children with exacerbations as compared to those with stable disease. It followed the trend demonstrated for the CRP level, which in the current study was significantly higher in children with exacerbations than in those with the stable disease, as previously shown by other authors [11].

It should be noted that the study by Uijterschout et al. was the only one to evaluate serum hepcidin levels in 49 children with cystic fibrosis, which makes it particularly important for comparisons [11]. In their study, the study group presented with significantly higher hepcidin levels as compared to controls, and—additionally—serum hepcidin levels were significantly higher in patients with exacerbations than in those with stable disease. Similar findings between the current and their study may stem from applying similar diagnostic criteria for exacerbation (i.e., elevated CRP level > 10 mg/L and/or worsening of bronchopulmonary disease in the last month requiring antibiotics). Children with exacerbations were older, had worse lung function parameters, and were more often tested positive for *P. aeruginosa*. The observed differences, on the other hand, can be explained by different hepcidin assay methods used in both studies. Uijterschout et al. used the technique of mass spectrometry (MS) in 9 cases (25%), which gave the result below the sensitivity threshold of the assay (<0.5 nM), whereas all those children presented with iron deficiency [11]. The current study utilised a competence ELISA assay for determination of the serum hepcidin level [23]. Although there are reports showing that hepcidin concentrations obtained using ELISA are up to three times higher compared to those obtained using the MS method, these values correlate [20, 24]. Lack of the standardized determination method also leads to statistically significant differences in measurement results obtained by different laboratories.

Hepcidin concentrations in the current study group were lower than those obtained by Gifford et al. in a group of 12 adults with cystic fibrosis [18]. The difference can be explained by the fact that the children were mostly in a good general condition in early stages of bronchopulmonary disease. The mean age of patients in the study by Gifford et al. was 32 years, and these were patients with advanced bronchopulmonary disease, meeting the exacerbation criteria. Gifford et al. assessed hepcidin and IL-6 levels in patients with exacerbations, before and after intravenous antibiotic therapy, observing a reduction of these parameters in response to treatment. Interestingly, even after treatment, serum hepcidin levels in their study group were higher than in paediatric patients enrolled in the current study (including those in the exacerbation phase). This may suggest that hepcidin levels increase as the patient ages and disease advances, probably due to continuous liver stimulation by inflammatory cytokines, especially IL-6, released to and present in the bloodstream. Higher serum hepcidin levels in adults with cystic fibrosis reflect the ongoing inflammatory process, more severe than the one seen in their relatively healthy paediatric counterparts. Indeed, higher concentrations of hepcidin, a peptide known for its antibacterial properties, reported in adults reflect bodily defence mechanisms triggered in response to infection with pathogens of higher virulence than those usually encountered in children. Another likely explanation is the loss of the ability of damaged liver cells to inhibit the synthesis of hepcidin in response to hypoxia and increased erythropoiesis. It is also possible that a gradual iron accumulation within the respiratory tract becomes a source of false information about normal or even elevated bodily iron stores and triggers increased hepcidin synthesis in the liver. A study to evaluate long-term changes in hepcidin synthesis and serum levels in patients with cystic fibrosis could provide plenty of important information about the exogenous and endogenous determinants of iron homeostasis in this group of patients.

The lack of standardized, age-appropriate reference standards limits the use of hepcidin as a marker of iron status in clinical practice. In 2013, Cangemi et al. attempted to determine the reference standards for healthy paediatric population. Hepcidin levels were determined in 86 children using the same ELISA kit, which was used in the current study and the reference range of 13.6–68 ng/mL (mean 40.8 ± 13.9 ng/mL) was suggested [25]. In the current study, the mean serum hepcidin levels in both the study group and healthy controls were below the proposed threshold, and the actual values remained below the threshold in almost half of the patients. In 2014, another report was published to propose reference ranges for serum hepcidin levels in children aged 0.5–3 years [20]. It suggested the ranges of 1.9–28.6 nmol/L and 0.6–13.9 nmol/L (conversion 1 nmol/L = 2789 *μ*g/L) as normal values for hepcidin levels determined using ELISA and MS, respectively. Hepcidin levels in the current study were, therefore, within the age norm according to that report. Furthermore, unlike Cangemi et al., who demonstrated that the hepcidin level in adolescent girls is higher as compared to that in adolescent boys, the study in question found no correlation between the hepcidin level and patient gender or age [25].

In the study in 22 adult patients with advanced cystic fibrosis, Gifford et al. demonstrated a correlation between the concentration of hepcidin and the severity of bronchopulmonary disease assessed using the Akron pulmonary exacerbation score [18]. This finding indicates the role of hepcidin as an acute phase protein involved in response to infection and/or inflammation. A positive correlation between hepcidin and CRP levels demonstrated in the current study is in keeping with the current knowledge about the regulation of hepcidin synthesis and with the results reported by other authors, including Galesloot et al., who showed such strong association in both adult men and women [22]. Furthermore, this finding makes the use of hepcidin as a marker of iron deficiency in patients with CF doubtful, as elevated hepcidin levels may reflect not only iron deficiency but also infections and chronic inflammation. Increased hepcidin levels during active inflammation suggest potential for using this parameter in early detection of exacerbations and monitoring treatment in patients with cystic fibrosis. In the current study, the suggested cutoff value for the hepcidin level, above which the risk of exacerbation increased markedly, was 17.19 ng/mL. The sensitivity and specificity of the test were 71% and 61%, respectively, which is still unsatisfactory, especially in comparison with those of conventionally used inflammatory markers. Other laboratory markers of inflammation include ESR, CRP, WBC count, PLT count, and haemoglobin assay. Among those markers, the C-reactive protein is the most known and relevant, due to its short half-life, quick elevation in line with the inflammatory response, and equally quick decrease as the inflammation resolves. Furthermore, CRP assay is cheap, easy, and commonly available. In the studied children with exacerbated CF, the CRP was significantly higher than that in children with stable disease, which is also confirmed by other studies [11]. Currently, serum hepcidin assay is not routinely used in diagnostic management, due to its significant price and availability limited to larger, university, and research centres. Hence, in order to evaluate the use of hepcidin in early detection of exacerbations and treatment monitoring, further research is required.

Iron deficiency as per WHO criteria could only be diagnosed in the current study sample in 2 (4%) well-nourished boys aged 9 and 21 months with stable disease. Interestingly, *P. aeruginosa* infection was confirmed in both cases despite the patients' young age. The apparent decreased incidence of iron deficiency in children with cystic fibrosis is associated with improved standards of medical care and an increased focus on appropriate nutrition, which significantly changed the natural history of the disease over the past few decades, mainly due to early diagnosis and treatment. Hepcidin levels in these patients were close to the lower threshold value, but a small group size made it impossible to carry out reliable statistical analysis. On the other hand, a small number of iron deficiency cases in the study group may be a mere underestimation. Ferritin is an acute phase protein, and its levels increase during inflammation, so higher values may as well indicate the severity of inflammation rather than improving iron status. It is particularly likely, as the number of patients with exacerbation in the study group was relatively high (*n* = 18; 39%). Within the study group, the mean ferritin level in children with exacerbations was only slightly higher than that in children with stable disease and the difference was insignificant, which is consistent with the findings of other authors [2, 7]. Considering the comparable results of ferritin concentrations in both groups and a wide range of values, it is doubtful that infection and inflammation were only reasons for elevated ferritin levels in children with cystic fibrosis, especially those with stable disease. Indeed, the interpretation of the concentrations of ferritin (<12 *μ*g/L) in the context of disturbances of iron in the cystic fibrosis leaves many doubts in both children and adults.

A significant limitation of the current study is the small sample size (especially the small number of children with iron deficiency) and group heterogeneity in terms of age and clinical manifestation. It warrants further research in a larger sample. Even though, it can be concluded that the serum hepcidin level appears a promising biomarker for assessing iron status in children with cystic fibrosis, especially those with exacerbated bronchopulmonary disease. It can also be useful in early detection and monitoring the treatment of exacerbations.

## Figures and Tables

**Table 1:** Hepcidin levels in the study and control groups.

	Range	Mean (SD)
Study group	3.61–55.19 *μ*g/mL	12.5 ± 10.5 *μ*g/mL
Control group	5.2–41.7 *μ*g/mL	11.8 ± 7.6 *μ*g/mL

**Table 2:** ROC metrics for serum hepcidin levels in the study group.

Test parameter	Value
Cutoff value	17.19 *μ*g/mL
AUC	0.7
Sensitivity	61%
Specificity	71%
PPV	58%
NPV	74%

## Data Availability

The data used to support the findings of this study are available from the corresponding author upon request.
